# Guest Editorial: Reflections on Medical Physics Training

**DOI:** 10.1120/jacmp.v13i4.4010

**Published:** 2012-07-05

The process of tightening qualifications to sit for ABR certification exams in subspecialties of medical physics is reaching the end of its arc. Predictably, this has sparked renewed hue and cry from individuals who would prefer to be offered lucrative careers without being held to those higher standards, as evidenced by a recent discussion thread on the electronic networking site LinkedIn. I offered the following perspective.

Medical physics is a fantastic career for people who have the requisite set of interests and skills. Where else do you get to be the master of such a diverse and complex set of high‐technology devices and systems, and do so directly in the cause of healing and comforting people with life‐threatening diseases? Not to mention earning the respect accorded to a Medical Specialist.

We each have to decide for ourselves at certain points in our lives what we want to do with ourselves, what our life priorities are, and what dues we are willing to pay to achieve our goals. In almost every profession, more people will enter the pipeline than will find life‐long careers coming out the other end. The good and the lucky succeed; others wind up doing something else. It's true for plumbers, ministers, English professors, and medical physicists. Nobody is entitled to a job by virtue of entering a degree program.

The profession of medical physics has changed dramatically since I entered 30 years ago. When I started out, nobody knew about this field — there was no pathway — and those of us who were lucky enough to find it (mostly by the good fortune of taking a wrong turn) came from very diverse backgrounds and learned the trade on the job. That worked well enough for the time because the field was very small, meaning opportunities for on‐the‐job training were fairly limited mostly to organizations with outstanding mentors who made careers of training good clinicians. I, for example, earned my MS degree from a formal medical physics didactic program in 1981 and spent three years in the clinic at the University of Florida as a full time trainee, apprenticing under some of the best physicists and physicians in the field.

When the IMRT bubble hit around 2000, it created an enormous imbalance in the career pathway ecology. There was suddenly demand for a large number of plausibly qualified people to do the new, highly lucrative billable procedures that required physics effort. The floodgates opened and a very large number of people came into the profession without proper (or any) didactic or clinical training. The profession has suffered. Medical physicists do not enjoy the same level of professional respect as they did, and there are now a whole lot more crappy jobs than I recall ever being the case. I trace that change in part to the influx of people into the field who did not understand the culture of Medical Specialty, did not understand the role or rightful standing of the medical physicist, and who enabled the existence of those crappy jobs merely by occupying them. The recent American Board of Radiology (ABR) changes, of which I am highly supportive, are an inevitable response by the Medical Specialty community — from CMS through American Board of Medical Specialties (ABMS) to ABR and to us — to the ridiculous porousness of our credentialing. We were the only ABMS‐certified profession that did not require formal clinical residency. Clearly that kind of exception could not stand.

There is no point bellyaching about how there are not enough residency slots, or about how there is no guarantee of eventual ABR certification for a current college junior. That's the way things work. Those who set their jaw, do the years of course work well at a quality program, and compete successfully for a quality residency will be rewarded with entry to a fantastic career and, I believe, an improving profession with improving quality of service to our patients. Some who enter the race will lose. That's the way it works for anything worth competing for.

Medical physics, despite tremendous relative growth, is still a very small profession. As I'm fond of saying, there are individual churches in Charlotte that have more members than does the AAPM. As such, personal connections are still very important. The people who run residency programs know the folks who run graduate programs. And the folks with the best entry‐level clinical jobs know the residency directors. Probably the single most important thing a person wishing to enter this field can do is to find out which programs command the most respect from the decision makers downstream and find a way to get in that part of the pipeline. The first thing I ask in looking at the resume of a prospective employee is with whom they did their clinical training. There is a short list of mentors whose name alone pushes the applicant to the top of the list. I'm not alone in that. And this is not an unreasonable bar to set — undergrads who are serious about careers in other professions do the legwork to find out which law schools or medical schools or other professional schools and which postgraduate training will net them the best odds of a good first job.

So, please, stop looking for guarantees. There are going to be some really good jobs for the foreseeable future and they are going to be competitive to obtain. So … compete. Do your homework regarding programs; choose wisely; distinguish yourself; pay your dues by getting a CAMPEP‐accredited graduate degree, followed by a CAMPEP‐accredited residency, followed by ABR subspecialty certification. Understand that having distinguished yourself in another specialty does not satisfy the requirements of this specialty *because it is a specialty*. And you might want to have a Plan B in your back pocket.

I'll finish by restating a key point. The very same changes that are constricting unqualified entry to medical physics jobs will make the profession more robust in the future. A profession that cannot demonstrate to a lay observer that its members have special skills earned through highly formalized and regulated education and training does not amount to much of a profession. That first job may look like the ultimate goal to someone just about to enter the pipeline but trust me, a job is just a job — but a professional career is for life.

George W. Sherouse, PhD, DABR, FAAPM

